# Circulating rotaviral RNA in children with rotavirus antigenemia

**DOI:** 10.1186/1477-5751-12-5

**Published:** 2013-02-01

**Authors:** Kamruddin Ahmed, Gulendam Bozdayi, Marcelo T Mitui, Selim Ahmed, Luthful Kabir, Dalgic Buket, Ilknur Bostanci, Akira Nishizono

**Affiliations:** 1 Research Promotion Institute, Oita University, Yufu, Japan; 2 Department of Clinical Microbiology, Faculty of Medicine, Gazi University, Ankara, Turkey; 3 Department of Microbiology, Faculty of Medicine, Oita University, Yufu, Japan; 4 Department of Pediatrics, Institute of Child and Mother Health, Dhaka, Bangladesh; 5 Department of Pediatric Gastroenterology, Faculty of Medicine, Gazi University, Ankara, Turkey; 6 Department of Pediatrics, Ministry of Health Ankara Educational and Research Hospital, Ankara, Turkey

**Keywords:** Human, Rotavirus, Antigenemia, Cytokine, Viremia

## Abstract

**Background:**

Rotavirus antigenemia is a common phenomenon in children with rotavirus diarrhea, but information is scarce on aspects of this phenomenon, such as genotype specificity, presence of intact viruses and correlation between genomic RNA and antigen concentration. Such information may help in understanding rotavirus pathogenesis and eventually be useful for diagnosis, treatment and prevention.

**Methods and findings:**

Serum samples were collected from children who presented at hospitals with diarrhea. Antigenemia was present in 162/250 (64.8%) samples from children with rotavirus diarrhea. No specific rotavirus genotype was found to be associated with antigenemia. Rotavirus particles could not be found by electron microscopy in concentrated serum from children with high levels of antigenemia. In passaged rotavirus suspension a significant correlation (r = 0.9559; P = 0.0029) was found between antigen level and viral copy number, but no significant correlation (r = 0.001480; P = 0.9919) was found between antigenemia level and viral copy number in serum. When intact rotavirus was treated with benzonase endonuclease, genomic double-stranded (ds) RNA was not degraded, but when sera of patients with antigenemia were treated with benzonase endonuclease, genomic dsRNA was degraded, indicating genomic dsRNA was free in sera and not inside virus capsid protein.

**Conclusions:**

Antigenemia is present in a significant number of patients with rotavirus diarrhea. Rotavirus viremia was absent in the children with rotavirus diarrhea who participated in our study, and was not indicated by the presence of antigenemia. The significance of circulating rotavirus antigen and genomic dsRNA in serum of patients with diarrhea deserves further study.

## Background

Globally, every year rotavirus infects 114 million children and accounts for about 453,000 deaths mainly in developing countries [1,2]. RVA is a non-enveloped virus with a triple-layered capsid containing 11 segments of double stranded RNA genome. The nucleotide sequence of VP7 and VP4 characterize the G and P genotypes, respectively [3]. These proteins are used in a binary classification system, 27 G and 35 P types thus far has been identified [4]. In general G1 through G4 and G9 are the most common types causing human infection [5]. A variety of diseases have been found to be associated with rotavirus [6,7]. Detection of rotavirus antigen and/or RNA in the central nervous system, heart, liver, testes, kidneys, bladder, liver biopsy from infant with cholestatic disease, respiratory secretions, lung cells, microvasculatures of heart and serum of those patients have provided indirect evidences of rotavirus or its components as a causative agent [6]. How rotavirus or its components reach other parts of the human body from the gastrointestinal tract is not known. Saulsburry et al. first reported the presence of rotavirus antigenemia in immunodeficient children with chronic rotavirus diarrhea [8]. Early work suggested that rotavirus antigenemia was the result of host immunological defects [8], but recent reports suggest that rotavirus antigenemia is commonly observed in immunocompetent children with rotavirus diarrhea.

Therefore, antigenemia could be at the center of the pathogenesis of various extraintestinal infections with rotavirus. However, several key characteristics of this antigenemia are unknown, such as whether antigenemia is associated with certain genotypes of rotavirus, whether antigenemia consists of intact virus particles, or whether there is any correlation between the concentrations of virus particles and genomic RNA in serum. Information on all these points will assist our understanding of the pathogenesis of rotavirus infection and may open new avenues for diagnosis, treatment and prevention of the disease. The aim of the present study was therefore to obtain further information about the characteristics of rotavirus antigenemia.

## Results

### Prevalence of rotavirus antigenemia in patients with and without rotavirus diarrhea

A total of 250 serum samples were available from patients with rotavirus diarrhea, and antigenemia was detected in 162 (64.8%) of these. Of the 250 serum samples, 169 were from Bangladeshi children: in this group, antigenemia was detected in 123 (72.8%) samples. Eighty-one samples were from Turkish children, and antigenemia was detected in 39 (48.1%) of these samples. A total of 126 samples were available from patients with diarrhea caused by agents other than rotavirus: rotavirus antigenemia was detected in 37 (29.4%) of these samples. Of these 126 serum samples, 7 were from Bangladeshi children, of which 5 (71.4%) showed antigenemia and 119 were from Turkish children, of which 32 (26.9%) showed antigenemia.

### Electron microscopic observation of serum concentrates from patients with antigenemia

Viral particles with characteristics suggestive of rotavirus were not found in any of the samples. This result indicates that intact rotavirus particle is absent in the serum of patients with rotavirus antigenemia.

### Correlation between viral copy number and ELISA reactivity of cultured rotavirus and serum samples

**Figure 1:**
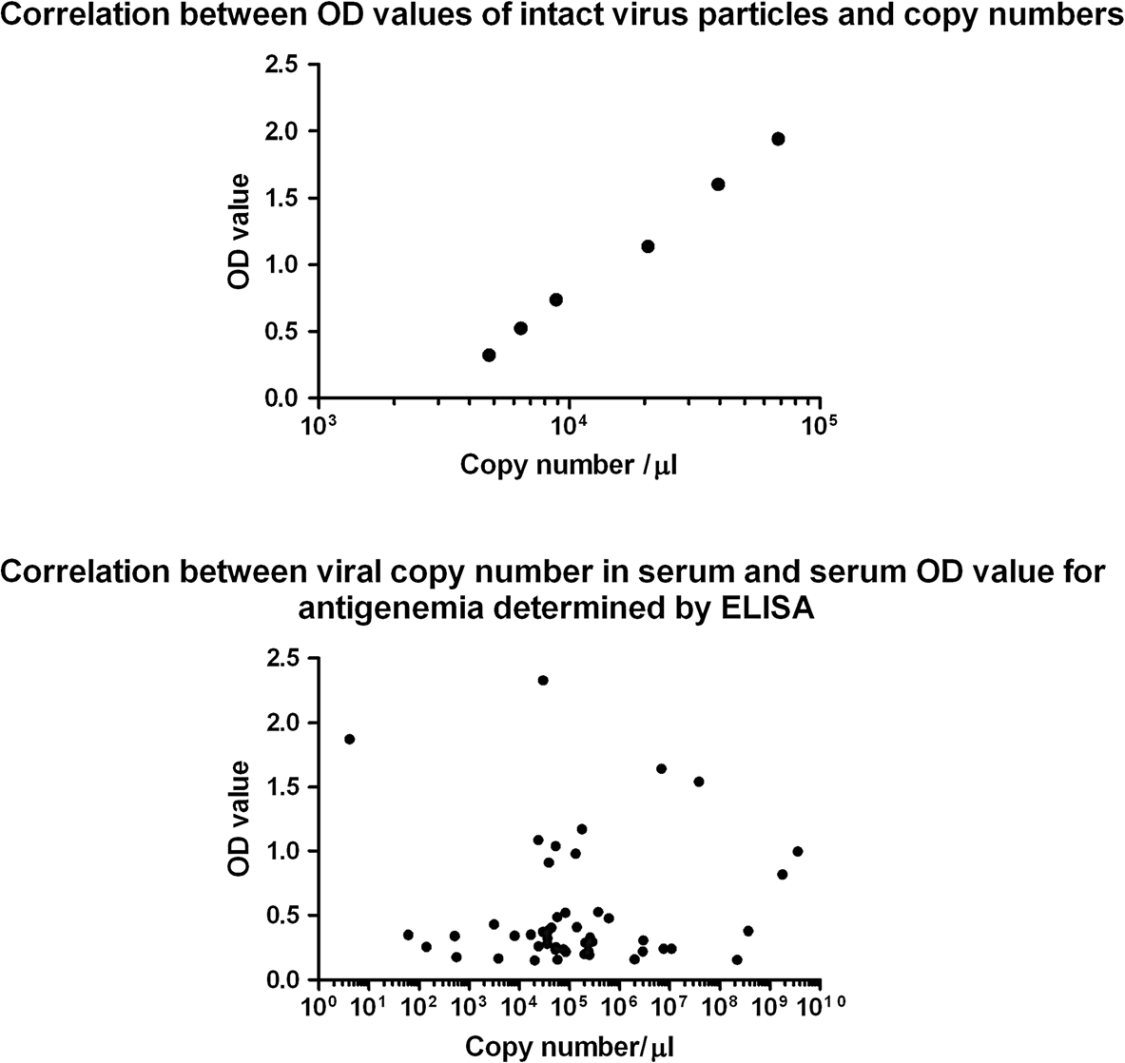
**Upper panel: Correlation between ELISA OD values of intact virus particles and VP6 gene copy numbers in RTqPCR.** Lower panel: Correlation between antigenemia determined by ELISA and VP6 gene copy number in serum.

Six samples containing different concentrations of rotavirus strain SA11 were used to determine whether there is a correlation between ELISA OD and the corresponding viral genomic copy number determined by RT-qPCR (Figure 1, Upper panel). The mean copy number and OD values were 2.47 × 10^4^ copies/μl (95% CI, 0 – 5.08× 10^4^ copies/μl) and 1.045 (95% CI, 0.3784 – 1.711), respectively. The correlation was significant: the Pearson r was 0.9559 (95% CI 0.6438 – 0.9953) and the *P* value was 0.0029. This result indicates that when intact rotavirus is present there is a significant correlation between OD and viral copy number, i.e. the intensity of the OD corresponds to the number of virus particles.

Serum samples from forty-nine patients were available to determine the correlation between antigenemia level determined by ELISA and the corresponding genomic copy number determined by RT-qPCR. Except one sample from a Turkish child all samples were rotavirus RNA positive. The mean copy number and OD values were 1.24 × 10^8^ copies/μl (95% CI, 0 – 2.89 × 10^8^ copies/μl) and 0.5264 (0.3866 – 0.6663), respectively. The correlation was not significant (Figure 1, Lower panel): the Spearman r was 0.001480 (95% CI −0.2878 – 0.2905) and the *P* value was 0.9919.

### Genomic RNA protection assay by benzonase endonuclease treatment

**Figure 2:**
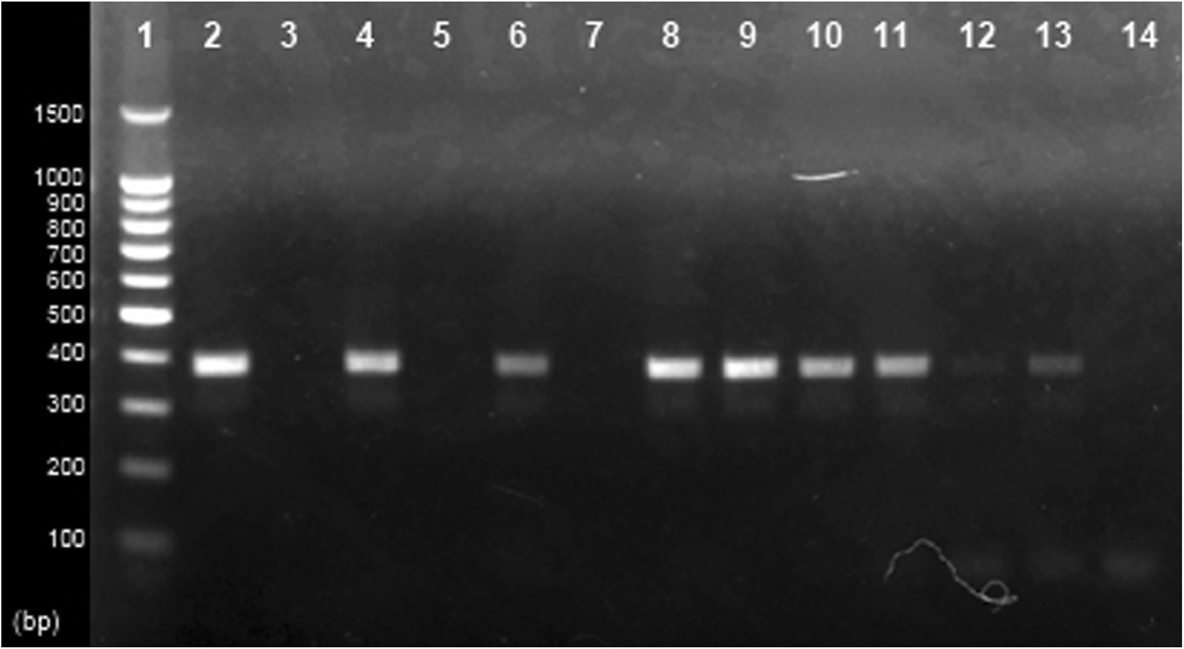
**Genomic RNA protection assay by treatment with benzonase endonuclease.** Agarose gel electrophoresis shows results of VP6 gene amplification by RT-PCR after treating genomic RNA and intact virus particles with benzonase endonuclease. Lane 1, 100 bp molecular weight marker; genomic RNA of different concentrations (7.3 × 10^7^, 9.0 × 10^5^ and 8.5 × 10^3^) of rotavirus SA11 strain were untreated (lanes 2, 4 and 6) and treated (lanes 3, 5 and 7) with 100 U of benzonase, different concentrations (7.3 × 10^7^, 9.0 × 10^5^ and 8.5 × 10^3^) of rotavirus SA11 strain were untreated (lanes 8, 10 and 12) and treated (lanes 9, 11 and 13) with 100 U of benzonase, and lane 14 contains negative control. Amplicons of VP6 (479 bp) are present in lanes 2, 4 and 6 and absent in lanes 3, 5 and 7, indicating that in these lanes, genomic RNA is degraded by benzonase endonuclease. Amplicons are present in lanes 8–13, indicating that genomic RNA is protected when intact rotavirus particles are treated with benzonase endonuclease.

**Figure 3:**
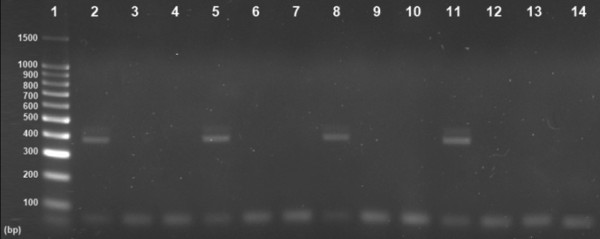
**Genomic RNA protection assay performed by treating sera of four antigenemia patients with benzonase endonuclease.** The photo of agarose gel electrophoresis shows an amplicon of the VP6 gene. Lane 1, contains a 100 bp molecular weight marker. In lanes 2, 5, 8 and 11, VP6 amplicons (479 bp) are present in RNA extracted from sera but not treated with benzonase endonuclease. In lanes 3, 6, 9 and 12, VP6 amplicons are absent in RNA extracted from sera then treated with benzonase endonuclease. In lanes 4, 7, 10 and 13, VP6 amplicons are absent in RNA extracted from sera that had been treated with benzonase endonuclease; and lane 14 contains negative control. The absence of amplicons of VP6 gene in sera treated with benzonase indicates that intact rotavirus particles were absent in sera of patients with antigenemia, and therefore RNA was degraded.

The results of the RT-PCR showed that extracted genomic RNA of rotavirus strain SA11 was completely degraded by benzonase nuclease, while untreated genomic RNA was intact and generated a 479 bp product in RT-PCR (Figure 2). When intact particles of rotavirus strain SA11 were treated with benzonase endonuclease, genomic RNA was not degraded and the VP6 gene could be amplified by RT-PCR from RNA extracted from these particles. When serum samples were treated with benzonase endonuclease and RNA was extracted, the VP6 gene could not be amplified by RT-PCR (Figure 3), while the VP6 gene could be amplified from RNA extracted from untreated sera. This assay provided evidence that naked RNA rather than intact virus particles were present in serum during rotavirus antigenemia.

### Distribution of VP7 genotypes in patients with and without antigenemia

**Figure 4:**
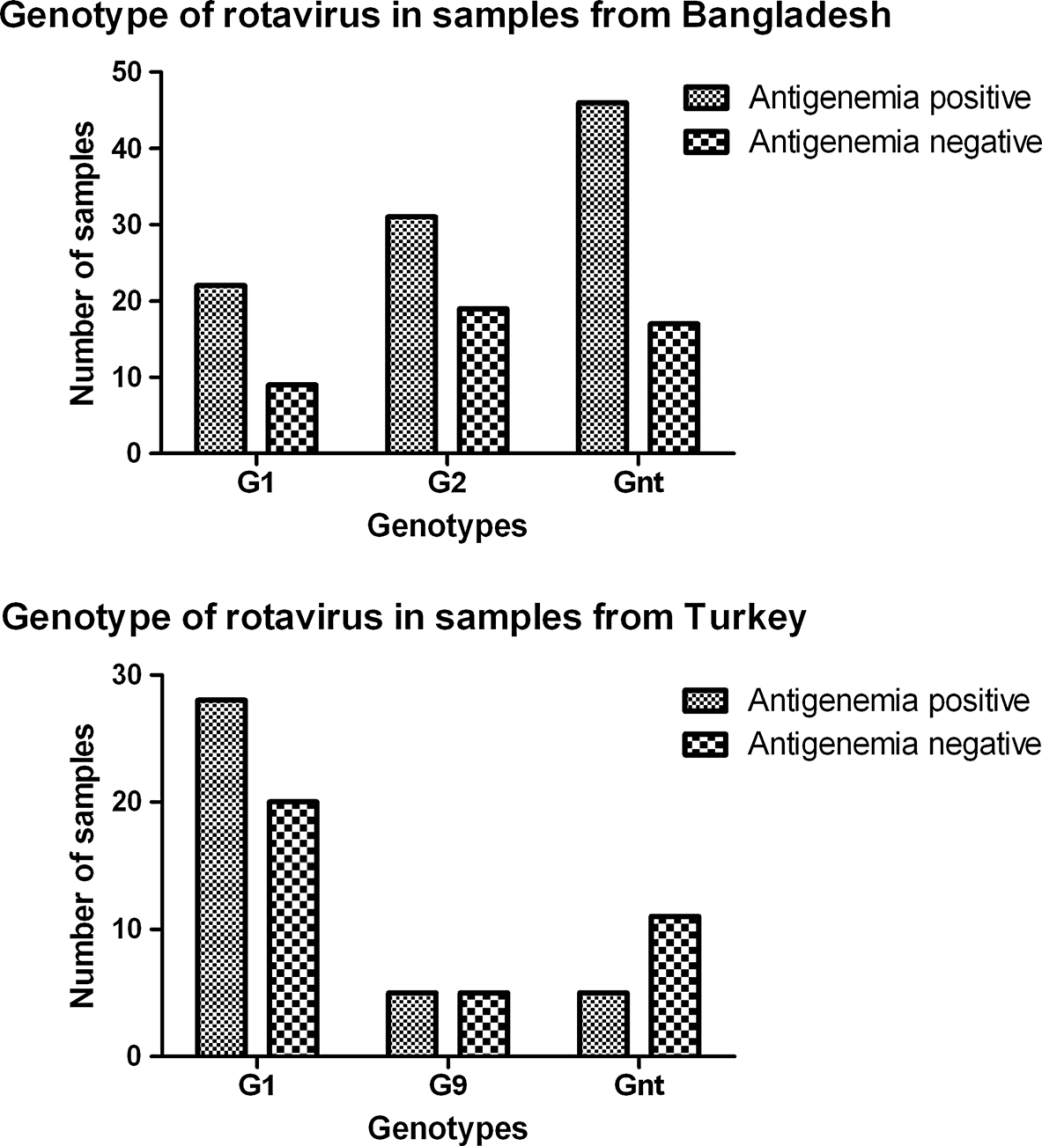
Distribution of rotavirus genotypes in Bangladeshi (upper panel) and Turkish children (lower panel) with and without antigenemia.

In Bangladeshi children, genotypes G1, G2, G4, G9, G12 and Gnt were found in 22 (18.5%), 31 (26.0%), 1 (1.8%), 8 (6.7%), 11 (9.2%) and 46 (38.6%) children with antigenemia, and in 9 (18.4%), 19 (38.8%), 1 (2.0%), 0, 3 (6.1%) and 17 (43.7%) children without antigenemia, respectively. In Turkish children, genotypes G1, G2, G3, G4, G9, and Gnt were found in 28 (71.8%), 0, 1 (2.6%), 0, 5 (12.8%) and 5 (12.8%) children with antigenemia, and in 20 (51.2%), 1 (2.6%), 1 (2.6%), 1 (2.6%), 5 (12.8%) and 11 (28.2%) children without antigenemia, respectively. A χ^2^ test was done to determine whether genotype distribution differed significantly between children with and without antigenemia. In Bangladeshi children, the following results were obtained for genotypes G1, G2 and Gnt: χ2 = 1.665, degrees of freedom (df) = 2 and *P* = 0.4350 (Figure 4). In Turkish children, the following results were obtained for genotypes G1, G9 and Gnt: χ2 = 3.532, df = 2 and *P* = 0.1710 (Figure 4). There was no significant difference in genotype distribution between children with and without antigenemia in either group.

## Discussion

The frequency of antigenemia in children with rotavirus diarrhea that was determined in the present study is within the range observed in other studies [9-13]. Although the prevalence of rotavirus diarrhea in Bangladesh and Turkey is similar [14,15], it is difficult to say why in Bangladeshi children the frequency of antigenemia was higher than Turkish children. Detection of rotavirus antigenemia is influenced by several factors such as increased acute-phase serum immunoglobulin G antibody titer [11], days after onset of diarrhea [11,12] and stool viral antigen levels [11]. Although in this study the frequency of antigenemia found in the rotavirus-negative diarrhea patients seems to be high compared with previous studies [13,16], comparison is difficult as studies on rotavirus antigenemia in children with rotavirus-negative diarrhea are scarce. The high frequency found in this study may reflect factors such as differences in study population, the high prevalence of rotavirus diarrhea in the study children or sensitivity of the method utilized to detect antigenemia. It is possible that the study children had had a rotavirus infection and recovered before collection of serum but continued to have diarrhea for some reason, because although antigen levels peak 1–3 days after the onset of symptoms and the serum of some children remains positive beyond 1 week, a decline in serum antigen values to baseline over a 3–4 week interval has been observed [11]. Rotavirus antigenemia has even been found in children with no gastroenteritis [10]: a recent study revealed that 18.5% of the healthy children have rotavirus in their stool [17]. Therefore, detecting several cases of rotavirus antigenemia in children without rotavirus diarrhea is not uncommon. In the present study the total number of such samples from Bangladeshi children was small and therefore the high positive antigenemia rate in those children can be almost ignored. Reviewing the serum sample collection period of rotavirus negative Turkish children showed that the majority of the antigenemia positive samples were from the rotavirus season which might be responsible for high frequency of antigenemia in this group of children.

In the case of rotavirus grown in the MA104 cell line, there may only be one infectious virus particle in 46,000 virus particles [18]. If there are a low number of infectious virus particles during rotavirus antigenemia, then serum culture will be unsuccessful. In the present study, two different approaches were undertaken to determine whether intact rotavirus is present in the serum of patients with antigenemia. In one approach, serum samples with high levels of antigenemia were observed under the electron microscope to obtain direct evidence of the presence of intact rotavirus particles. In the other approach, we predicted that the presence of intact rotavirus particles in serum samples of patients with antigenemia will protect genomic RNA from endonuclease and there will be a correlation between serum antigen level and viral copy number. Benzonase endonuclease was used for the endonuclease protection assay. Benzonase endonuclease degrades both DNA and RNA, whether single-stranded, double-stranded, linear, circular or supercoiled (http://wolfson.huji.ac.il/purification/PDF/DNA_Removal/NOVOGEN_Benzonase.pdf).

The use of electron microscopy in serological identification of viruses is an established technique. Two major limitations of electron microscopic detection of extracellular viruses are the requirement for a relatively high concentration of virus (10^5^ to 10^7^/ml, depending on size) and the presence of background materials (such as serum proteins) that may mask the virus particles [19]. The concentrations of virus in our samples were more than adequate to be observed under the electron microscope. The ELISA OD values of the serum samples used for electron microscopy correspond to more than 5 × 10^5^ rotavirus particles/ml. In addition, the samples were concentrated 20× concentrated by ultracentrifugation, which should make the concentration of any viral particles present in the serum at least 1 × 10^7^ rotavirus particles/ml. Had there been viremia during rotavirus diarrhea, these serum samples would have contained enough rotavirus particles to be visualized by electron microscopy. Our experience with cultured rotavirus particles indicates that the characteristic features of rotavirus particles are unlikely to be missed by electron microscopy, even in the presence of background materials, because they cannot mask all the virus particles.

In this study, we provided evidence that the ELISA OD of intact virus correlates significantly with the copy number. However, serum ELISA OD values did not correlate with serum virus copy numbers, which suggested that intact virus particles are not present during rotavirus antigenemia. The absence of intact virus particles in the serum of patients with antigenemia was confirmed by showing degradation of viral RNA in serum by benzonase endonuclease treatment. In an intact virus particle, the protein coat protects the genome from degradation by nucleases, therefore absence of this protection during treatment with benzonase endonuclease demonstrated that viremia was absent during rotavirus diarrhea.

There is only one study that showed the presence of viremia in all of the antigenemia-positive and 22% of the antigenemia-negative children tested by culturing their sera [10]. Virus culture may be more sensitive than electron microscopy, but PCR after benzonase treatment of serum as used in this study is more sensitive than virus culture. The present study provides evidence that free viremia is absent in patients with rotavirus antigenemia, possibly indicating a role for differences in population genetics in causing viremia.

Previous results confirmed that rotavirus G genotypes of the fecal and serum samples from the same patient were identical [20]. Therefore, we used the genotypes found in stool as representative of the genotypes causing antigenemia in the corresponding serum samples. We found no significant differences in genotype distribution between antigenemia positive and negative patients, indicating that it is unlikely that particular genotypes are capable of causing antigenemia. Similar results were found by Fujita et al. [20], which together with our data suggests that there is no particular genotype of rotavirus that is more likely to cause antigenemia.

In conclusion, there was no specific rotavirus genotype associated with antigenemia, and viremia was absent in children with rotavirus antigenemia. Further study is needed to verify whether there is cell-associated viremia in children with rotavirus antigenemia.

## Materials and methods

### Ethics review of the proposal and consent

The research proposal was approved by the ethics review board of the Faculty of Medicine, Gazi University and the ethics committee of the Institute of Child and Mother Health (ICMH). The verbal consent of the child’s guardian was obtained prior to sample collection. Since a fraction was obtained from samples sent to the laboratory of routine diagnostic purposes therefore informed written consent was not obtained and it was not required by the ethics review boards.

### Collection of specimens

Children enrolled in this study were less than 5 years old. This is part of studies on rotavirus diarrhea previously carried out in Turkey and Bangladesh [14,15]. Between September 2004 and December 2005, samples were collected from patients who attended at Gazi University Hospital and the Ministry of Health Ankara Educational and Research Hospital in Ankara, Turkey with watery diarrhea. During July 2005-June 2006, samples were collected from children who attended at the affiliated hospital of ICMH, Matuail, Dhaka, Bangladesh with watery diarrhea. A case of diarrhea was defined as passing looser than normal stool three or more times, during a 24 hour period. One stool and one serum sample were collected from each patient and stored at −80°C until use.

### Detection of rotavirus in stool

Rotavirus antigen was detected from a 10% stool dilution in phosphate-buffered saline by enzyme immunoassay (Rotaclone, Meridian Diagnostics Inc., Cincinnati, Ohio, USA), according to the manufacturer’s instructions.

### Detection of rotavirus antigenemia

Rotavirus antigenemia was detected by a previously published method [21]. A twofold dilution of serum was made with phosphate-buffered saline (pH 7.2), and 100 μl of this was used for rotavirus antigen detection by Rotaclone. Absorbance of 0.15 or more was used as the criterion for positive reactions.

### Observation of serum concentrates under electron microscopy

Four serum samples with relatively high optical density (OD) were selected during the detection of antigenemia for observation by electron microscopy. Samples 184 and 109 (OD values 2.333 and 1.874) were obtained from two Bangladeshi children with rotavirus diarrhea and antigenemia. Samples AHP20 and GUP30 (OD values 0.409 and 0.373) were obtained from two Turkish children with rotavirus diarrhea and antigenemia. Serum was concentrated by ultracentrifugation: 400 μl of serum was mixed with an equal volume of 0.9% NaCl solution and laid on a 30% sucrose cushion. Using a Beckman 100.2 rotor, samples were centrifuged at 50,000 rpm for 1 hour at 4°C. The pellet was resuspended in 20 μl 0.9% NaCl solution, and 5 μl of this was applied to a carbon-coated copper grid and then stained with 2% uranyl acetate for 30 sec. The excess of fluid was removed by a piece of filter paper. Specimens were observed under a JEM 100C electron microscope (JEOL Ltd., Tokyo, Japan) operated at 80 kV. The grids were observed blinded at a magnification of × 36,000 or greater [22].

### Correlation between ELISA OD and copy number of cultured virus

To determine whether there is a correlation between ELISA OD and virus genome copy number when intact virus particles are present in a fluid, cultured rotavirus strain SA11 suspended in phosphate-buffered saline (PBS) was serially diluted twofold in PBS, and then ELISA OD was determined as described above and the copy number in each diluted sample was determined as described below. The correlation between ELISA OD and copy number was determined as described in the statistical analysis section.

### Determination of virus copy number in serum

Rotavirus copy number was determined by reverse transcription quantitative polymerase chain reaction (RT-qPCR) [23]. Nucleic acid was extracted from 140 μl of serum using the QIAamp Viral RNA Mini kit (Qiagen Co. Ltd., Tokyo, Japan). RT-qPCR with VP6-specific primers was carried out using the QuantiTect SYBR Green RT-PCR kit (Qiagen) and a LightCycler 480 (Roche, Mannheim, Germany) real-time PCR thermal cycler. The primers VP6- F (sense) (5′ − GACGGVGCRACTACATGGT − 3′) (nucleotides [nt] 747–766) and VP6-R (antisense) (5′ − GTCCAATTCATNCCTGGTGG − 3′) (nt 1126–1106) amplified a 379-bp region (nt 747–1126) of the VP6 gene [24]. The genomic dsRNA was extracted from cultured rotavirus strain SA11 by the phenol − chloroform − isoamyl alcohol method and run on a 0.8% agarose gel. Segment 6 was cut out, and then dsRNA was extracted and purified by QIAquick Gel Extraction kit (Qiagen) according to the manufacturer’s instructions. A standard curve was generated by amplifying a known amount of dsRNA [25]. One copy of rotavirus equals 1.29 × 10^−12^ μg of segment 6 dsRNA. A serial 10-fold dilution of extracted and purified VP6 was used for calibration using the LightCycler 480 software and a reference gene for normalization between PCR runs. The crossing point, defined as the PCR cycle at which the amplification signal intersects the log linear region, was used as a direct correlate of the amount of rotavirus RNA present in the serum. Each sample was measured three times and the average was taken to calculate the copy number.

### Genomic RNA protection assay by benzonase endonuclease treatment

To determine whether antigenemia consists of intact rotavirus particles, sera were treated with benzonase endonuclease (Novagen EMD Chemicals, San Diego, CA, USA), and then VP6-specific RT-PCR was performed to determine the presence or absence of genomic RNA. In the presence of intact virus particles, the genomic RNA will not be degraded by endonuclease and the target gene will be amplified by RT-PCR. However, if naked RNA is present, it will be degraded and RT-PCR will show a negative result. To determine the optimum conditions for this assay, different amounts (7.3 × 10^7^, 9.0 × 10^5^ and 8.5 × 10^3^) of rotavirus SA11 strain and corresponding genomic RNA in distilled water were treated with 100 U of benzonase for 22 hours at 37°C according to the manufacturer’s instructions. The reaction was stopped by adding EDTA at a final concentration of 2 mM to inactivate by the benzonase. Specimens handled similarly without benzonase treatment were used as a control. Nucleic acid was extracted using a QIAamp viral RNA minikit according to the manufacturer’s instructions and subjected to RT-PCR for the detection of the VP6 gene using primers and methods described elsewhere [14,24].

### Distribution of genotypes in patients with and without antigenemia

To determine the effects of rotavirus genotype distribution on antigenemia, the genotype distribution of rotavirus found in stool was compared in patients with and without antigenemia. Genotypes were determined by RT-PCR as described in our previous studies [14,15].

### Statistical analysis

The Mann–Whitney test and Spearman rank correlation, appropriate for experimental data that display a non-Gaussian distribution [26], were used to calculate two-tailed P values. The Pearson correlation was used for experimental data that displayed a Gaussian distribution. The χ^2^ test was used to determine whether genotype distribution differed between patients with and without antigenemia. All statistical analyses were performed using Prism 5 software (GraphPad Software Inc., La Jolla, CA, USA).

## Competing interests

The authors have declared that no competing interest exists.

## Authors’ contributions

ICMJE criteria for authorship read and met: KA, GB, MTM, SA, LK, DB, IB, and AN. Agree with the manuscript’s results and conclusions: KA, GB. MTM, SA, LK, DB, IB, and AN. Conceived the idea, designed the study, and secured funds for the study: KA. Analyzed the data, did statistical analyses and wrote the manuscript: KA. Contributed in experiment plan and provided technical assistance: KA, GB, MTM and AN. Contributed in the study plan, enrolled patients, obtained verbal consent, collected clinical samples and demographic data, and maintained clinical samples: SA, LK, DB, IB. Coordinated the project: KA, GB and SA. Contributed to interpretation of data, writing of the manuscript: KA, GB, MTM, SA, LK, DB, IB, and AN. All authors read and approved the final manuscript.

## References

[B1] Tate JE Burton AH Boschi-Pinto C Steele AD Duque J 2008 estimate of worldwide rotavirus-associated mortality in children younger than 5 years before the introduction of universal rotavirus vaccination programmes: a systematic review and meta-analysis Lancet Infect Dis 2012 12 136 141 10.1016/S1473-3099(11)70253-5 22030330

[B2] Chandran A Fitzwater S Zhen A Santosham M Prevention of rotavirus gastroenteritis in infants and children: rotavirus vaccine safety, efficacy, and potential impact of vaccines Biologics 2010 4 213 229 20714358 10.2147/btt.s6530 PMC2921258

[B3] Greenberg HB Estes MK Rotaviruses: from pathogenesis to vaccination Gastroenterology 2009 136 1939 1951 10.1053/j.gastro.2009.02.076 19457420 PMC3690811

[B4] Matthijnssens J Ciarlet M McDonald SM Attoui H Banyai K Uniformity of rotavirus strain nomenclature proposed by the Rotavirus Classification Working Group (RCWG) Arch Virol 2011 156 1397 1413 10.1007/s00705-011-1006-z 21597953 PMC3398998

[B5] Glass RI Parashar UD Bresee JS Turcios R Fischer TK Rotavirus vaccines: current prospects and future challenges Lancet 2006 368 323 332 10.1016/S0140-6736(06)68815-6 16860702

[B6] Crawford SE Patel DG Cheng E Berkova Z Hyser JM Rotavirus viremia and extraintestinal viral infection in the neonatal rat model J Virol 2006 80 4820 4832 10.1128/JVI.80.10.4820-4832.2006 16641274 PMC1472071

[B7] Hung CW Wu WF Wu CL Rotavirus gastroenteritis complicated with toxic megacolon Acta Paediatr 2009 98 1850 1852 10.1111/j.1651-2227.2009.01444.x 19650837

[B8] Saulsbury FT Winkelstein JA Yolken RH Chronic rotavirus infection in immunodeficiency J Pediatr 1980 97 61 65 10.1016/S0022-3476(80)80131-4 6247473

[B9] Sugata K Taniguchi K Yui A Miyake F Suga S Analysis of rotavirus antigenemia and extraintestinal manifestations in children with rotavirus gastroenteritis Pediatrics 2008 122 392 397 10.1542/peds.2007-2290 18676558

[B10] Blutt SE Matson DO Crawford SE Staat MA Azimi P Rotavirus antigenemia in children is associated with viremia PLoS Med 2007 4 e121 10.1371/journal.pmed.0040121 17439294 PMC1852122

[B11] Fischer TK Ashley D Kerin T Reynolds-Hedmann E Gentsch J Rotavirus antigenemia in patients with acute gastroenteritis J Infect Dis 2005 192 913 919 10.1086/432549 16088842

[B12] Blutt SE Kirkwood CD Parreno V Warfield KL Ciarlet M Rotavirus antigenaemia and viraemia: a common event? Lancet 2003 362 1445 1449 10.1016/S0140-6736(03)14687-9 14602437

[B13] Ramani S Paul A Saravanabavan A Menon VK Arumugam R Rotavirus antigenemia in Indian children with rotavirus gastroenteritis and asymptomatic infections Clin Infect Dis 2010 51 1284 1289 10.1086/657069 21039217

[B14] Ahmed K Ahmed S Mitui MT Rahman A Kabir L Molecular characterization of VP7 gene of human rotaviruses from Bangladesh Virus Genes 2010 40 347 356 10.1007/s11262-010-0463-x 20217207

[B15] Bozdayi G Dogan B Dalgic B Bostanci I Sari S Diversity of human rotavirus G9 among children in Turkey J Med Virol 2008 80 733 740 10.1002/jmv.21120 18297696

[B16] Ray P Fenaux M Sharma S Malik J Subodh S Quantitative evaluation of rotaviral antigenemia in children with acute rotaviral diarrhea J Infect Dis 2006 194 588 593 10.1086/505878 16897656

[B17] Ayukekbong J Lindh M Nenonen N Tah F Nkuo-Akenji T Enteric viruses in healthy children in Cameroon: viral load and genotyping of norovirus strains J Med Virol 2011 83 2135 2142 10.1002/jmv.22243 22012721

[B18] Ward RL Knowlton DR Pierce MJ Efficiency of human rotavirus propagation in cell culture J Clin Microbiol 1984 19 748 753 6088569 10.1128/jcm.19.6.748-753.1984 PMC271178

[B19] Lee FK Nahmias AJ Nahmias DG McDougal JS Demonstration of virus particles within immune complexes by electron microscopy J Virol Methods 1983 7 167 181 10.1016/0166-0934(83)90006-X 6315751

[B20] Fujita Y Liu B Kohira R Fuchigami T Mugishima H Rotavirus antigenemia and genomia in children with rotavirus gastroenteritis Jpn J Infect Dis 2010 63 83 86 20332567

[B21] Nakagomi T Nakagomi O Rotavirus antigenemia in children with encephalopathy accompanied by rotavirus gastroenteritis Arch Virol 2005 150 1927 1931 10.1007/s00705-005-0565-2 15959833

[B22] Brandt CD Kim HW Rodriguez WJ Thomas L Yolken RH Comparison of direct electron microscopy, immune electron microscopy, and rotavirus enzyme-linked immunosorbent assay for detection of gastroenteritis viruses in children J Clin Microbiol 1981 13 976 981 6263947 10.1128/jcm.13.5.976-981.1981 PMC273926

[B23] Kang G Iturriza-Gomara M Wheeler JG Crystal P Monica B Quantitation of group A rotavirus by real-time reverse-transcription-polymerase chain reaction: correlation with clinical severity in children in South India J Med Virol 2004 73 118 122 10.1002/jmv.20053 15042658 PMC2459214

[B24] Iturriza Gomara M Wong C Blome S Desselberger U Gray J Molecular characterization of VP6 genes of human rotavirus isolates: correlation of genogroups with subgroups and evidence of independent segregation J Virol 2002 76 6596 6601 10.1128/JVI.76.13.6596-6601.2002 12050372 PMC136279

[B25] Ayala-Breton C Arias M Espinosa R Romero P Arias CF Analysis of the kinetics of transcription and replication of the rotavirus genome by RNA interference J Virol 2009 83 8819 8831 10.1128/JVI.02308-08 19553303 PMC2738170

[B26] Richardson BA Overbaugh J Basic statistical considerations in virological experiments J Virol 2005 79 669 676 10.1128/JVI.79.2.669-676.2005 15613294 PMC538531

